# Facilitators and barriers to optimal home blood pressure management in patients with hypertensive disorders of pregnancy in a tertiary care facility in Abuja, Nigeria: a qualitative research study

**DOI:** 10.1186/s12913-023-09976-6

**Published:** 2023-09-06

**Authors:** Zainab Mahmoud, Adaego A. Orji, Chukwuebuka F. Okoye, Friday O. Ameh, Erica Jamro-Comer, Aliyu Isah, Bissallah Ekele, Godwin Akaba, Dike B. Ojji, Mark D. Huffman

**Affiliations:** 1 Washington University, 660 S Euclid Ave, Campus, Box 6068, St Louis, MO 63110 USA; 2 Cardiovascular Research Unit, University of Abuja and University of Abuja Teaching Hospital, Gwagwalada, Abuja, Nigeria; 3 Faculty of Clinical Sciences, University of Abuja, Abuja, Nigeria; 4 The George Institute for Global Health, Sydney, Australia

**Keywords:** Hypertensive disorders of pregnancy, Pregnancy, Facilitators, Barriers, Home blood pressure monitoring program

## Abstract

**Background:**

Nigeria has one of the highest burdens of maternal deaths globally, and hypertensive disorders of pregnancy (HDP) are the leading cause of maternal morbidity and mortality in the country. There is a significant implementation gap in utilizing evidence-based practices for the management of HDP in Nigeria. This study evaluated facilitators and barriers to implementing a home blood pressure monitoring program to improve management of HDP.

**Methods:**

From August 2022 to September 2022, we conducted 15 semi-structured, key informant interviews and 4 focus group discussions among patients, health care workers, and administrators at University of Abuja Teaching Hospital (UATH), a tertiary care centre in Nigeria. The study used the Consolidated Framework for Implementation Research to assess five domains: individual characteristics, inner and outer settings, intervention characteristics, and process of implementation. Audio files were transcribed, and data were analysed using a combination of inductive and deductive approaches. We also conducted 32 brief surveys on the participants to assess acceptability, appropriateness, and feasibility of a blood pressure monitoring program.

**Results:**

The study sample consisted of healthcare workers (*n=*22) including specialists in cardiology, obstetrics and gynaecology, maternal-foetal medicine, nurses/midwives and resident doctors as well as patients (*n=*10). Mean (SD) age was 39.5 (10.9), and 78% were female. Participants identified facilitators including the perceived simplicity of home blood pressure monitoring program, high burden of HDP, and availability of a multi-disciplinary team of healthcare professionals with expertise in HDP management. Barriers identified were cost, limited knowledge of HDP amongst patients, limited transportation networks, inconsistent management protocols, and inadequate manpower and facilities. Survey results indicated that between 81% and 88% of participants reported that a blood pressure monitoring program would be acceptable, 56%-72% reported that it would be appropriate, and 47%-69% reported that it would be feasible.

**Conclusion:**

This study identified facilitators and barriers while highlighting key implementation strategies to leverage and effectively address these respectively to enable successful implementation of a home blood pressure monitoring program. It also demonstrated that a home blood pressure monitoring program was considered acceptable, appropriate and feasible among respondents interviewed at UATH.

## Background

Nigeria has one of the highest burdens of maternal deaths worldwide, with an estimated 1047 deaths per 100,000 live births accounting for 29% of all maternal deaths globally [1]. With this rate of maternal deaths, it is unlikely that Nigeria will achieve the United Nations Sustainable Development Goal (SDG) of decreasing maternal deaths to less than 70 per 100,000 live births by 2030. Urgent and effective implementation of evidence-based strategies to prevent, detect, treat, and control maternal complications are needed to reduce this large burden of disease [2].

Hypertensive disorders of pregnancy (HDP - chronic hypertension, gestational hypertension, preeclampsia, eclampsia, HELLP syndrome, and preeclampsia superimposed on chronic hypertension) are the leading cause of maternal morbidity and mortality in Nigeria. A 2022 study of 76,563 women admitted for delivery in a nationwide network of referral level hospitals found that HDP accounted for 32% of maternal deaths [3]. Most maternal deaths from HDP are potentially preventable with timely and effective care [4]. It is estimated that 50% of maternal deaths in Sub-Saharan Africa occur postpartum, when mothers’ care transition from obstetricians to other clinicians [5, 6]. This postpartum period, coined “the 4th trimester” is a critical time for monitoring and treating women at increased risk of cardiovascular disease complications, including cardiomyopathy, stroke, and postpartum eclampsia [7]. This period also provides an opportunity to implement a team-based care approach using a cardio-obstetrics model in implementing strategies to mitigate maternal morbidity and mortality and to improve outcomes [8].

Evidence-based strategies are being employed to reduce the impact of hypertensive disorders of pregnancy (HDP) in high-income countries, including postpartum blood pressure (BP) monitoring, elevated BP treatment, and comprehensive postpartum follow-up as suggested by the American College of Obstetricians and Gynaecologists and World Health Organization [9, 10]. However, the feasibility, acceptability, and effectiveness of these programs in a resource-limited, high-burden setting such as Nigeria is unclear.

Despite the high burden of HDP in Nigeria and available evidence-based practices, there remains a significant knowledge-to-action gap in the utilization of these practices. Successful implementation of evidence-based practices requires consideration of factors such as the selection of appropriate and acceptable interventions, identification of barriers and facilitators, adaptation to local contexts, and evaluation of implementation success or failure [11]. Thus, we sought to evaluate the facilitators and barriers of the current and optimal management of HDP with a focus on home BP monitoring in Nigeria using qualitative research methods. This will serve as formative research study to guide implementation of a home BP monitoring program.

## Methods

### Study design and setting

This was a qualitative study designed to understand the facilitators and barriers to optimal management of HDP at the University of Abuja Teaching Hospital (UATH), a tertiary care centre located in the Federal Capital Territory of Abuja, Nigeria. The hospital is a referral centre for pregnant patients in Abuja and neighbouring states.

The study used the Consolidated Framework for Implementation Research (CFIR) to assess five key domains: intervention characteristics, individual characteristics, inner setting, outer setting, and implementation process. The research team, comprised of the principal investigator and 2 research assistants, conducted 15 in-depth in-person interviews and 4 focus group discussions with 3-6 participants each comprised of physicians, nurses, midwives, patients, and administrators from UATH, between August 2022 to September 2022. The sample was selected using a maximal variability sampling frame approach with additional participants recruited through a snowball approach to achieve thematic saturation. The study was approved by the Institutional Review Board at the Washington University in St. Louis and UATH Health Research Ethics Committee.

### Data collection and analysis

Interviews were conducted by the principal investigator and 2 trained research assistants using a pre-designed interview guide that used CFIR to evaluate: individual characteristics, inner and outer settings, intervention characteristics, and process of implementation of a home BP monitoring program to optimize management of HDP in the hospital. All interviews were conducted in person, in English, audio recorded, and subsequently transcribed by an independent transcriber. Field notes were taken during the interview. Interview duration ranged from 10 minutes to 60 minutes.

A combination of inductive and deductive approaches was used for the analysis while applying key stages of framework analysis, including data familiarization and systematic coding using the CFIR 2.0 to interpret and condense data into themes. Dedoose version 9.0.78 software was used for data analysis. We followed the Consolidated Criteria for Reporting Qualitative Research (COREQ) guidelines to present the findings of this study [12]. To complement the qualitative data collection, participants completed a demographic questionnaire and an Acceptability of Intervention Measure (AIM) survey to assess feasibility, appropriateness, and acceptability of the home BP monitoring program [13]. The standard taxonomy of implementation outcomes developed by Proctor et al. served as a framework to guide the definition and assessment of acceptability, appropriateness, and feasibility within the context of implementing a home BP monitoring program [14].

Acceptability was defined as the perception among implementation stakeholders that a home BP monitoring program is agreeable, palatable, or satisfactory. Appropriateness was defined as the perceived fit, relevance, or compatibility of the home BP monitoring program at UATH and for postpartum patients with HDP. Feasibility was defined as the extent to which the home BP monitoring program can be successfully executed by patients with HDP at UATH. Descriptive statistics were used to summarize the participants characteristics and responses to survey questions.

## Results

### Participant characteristics

The study sample consisted of 32 individuals, comprising of healthcare workers (i.e., physicians, nurses, midwives, and administrators) and patients, all from UATH (Table 1). Physician participants included specialists in cardiology, obstetrics and gynaecology, maternal-foetal medicine, and resident doctors. Patient participants were women who were either pregnant or had recently given birth and were receiving care at either at the antenatal or postnatal clinics.

**Table 1:** Interview and Focus-Group Discussion Participant Characteristics

***Participant Characteristics***	**N (%)**
*Total*	32
*Role*	
* Physicians*	11 (34)
* Nurses/Midwives*	6 (19)
* Administrators*	5 (16)
* Patients*	10 (31)
*Gender*	
* Male*	7 (22)
* Female*	25 (78)
*Education*	
* Secondary*	1 (3)
* Lower or Higher Diploma*	4 (12)
* Undergraduate Degree*	13 (41)
* Postgraduate Degree*	14 (44)

We report themes of the facilitators and barriers to implementing a home BP monitoring program in the management of HDP, as well as strategies to overcome these barriers. Participants' responses covered various constructs from the updated CFIR domains and are shown in Table 2. These included 16 constructs that acted as facilitators (i.e., complexity, design, adaptability, self-efficacy, motivation, readiness for implementation, tension for change, planning, engaging, executing) and barriers (i.e., cost, knowledge and beliefs, structural characteristics, external policies, partnerships and connections, local conditions), and further described below.

**Table 2:** CFIR Domains and Constructs of Participants’ Responses of Facilitators and Barriers to Implementing a Home BP Monitoring Program

**CFIR DOMAIN AND CONSTRUCTS**	**FACILITATORS**	**BARRIERS**
**1. Innovation** a. Complexity b. Design c. Cost d. Adaptability	Home blood pressure monitoring is a simple intervention Intervention is appropriate for the setting	Financial burden on patients for seeking care Inadequate funds to sustain the project
**2. Individual Characteristics** Capacity a. Knowledge and beliefs b. Self-efficacy c. Motivation	Patients are capable of checking BP independently Patients feel motivated to participate in program	Sociocultural and religious beliefs about program and research Limited knowledge about HDP amongst patients
**3. Inner Setting** a. Readiness for implementation b. Compatibility c. Tension for change d. Structural Characteristics	HDP is prevalent and identified as an important area of focus Large referral center with expertise for management of HDP	Inadequate manpower and equipment in the hospital
**4. Outer Setting** a. External policies b. Local conditions c. Partnerships and connections	Availability of mobile telecommunications Health insurance schemes can be utilized to improve access to care	Accessibility to the hospital Lack of supportive government policies to expand care
**5. Process** a. Planning b. Engaging c. Executing	Leverage ANC to educate participants on program Protocols are commonly used in the hospital	Cumbersome processes in hospital can deter patients from attending clinics

*CFIR *Consolidated Framework for Implementation Research, *BP* Blood pressure, *HDP* Hypertensive disorders of pregnancy

### Facilitators

Participants’ highlighted important facilitators that could be leveraged to ensure successful implementation of the home BP monitoring program. These include:

1. *Home BP monitoring is perceived as a simple intervention (innovation)*

Participants perceived home BP monitoring as a simple intervention and felt most patients would be able to perform despite low literacy levels in the population.

*“The blood pressure apparatus is relatively easy to use. Most of the patients that access care here have at least secondary level education. I think teaching them to use the home blood pressure monitor should not be too much of a challenge.” (Cardiology Registrar)*

Additionally, some participants compared home BP monitoring with self-monitoring of blood glucose using glucometers, a patient-level intervention currently utilized at UATH in the management of diabetes.

*“I believe that they would make use of that BP apparatus because if you look at the patients that are diabetic patients, they have their own glucometer, we teach them how to use it and they are making very good use if it, you understand.” (Nurse, maternity ward)*

Patients who were pregnant or postpartum expressed their motivation to participate in a home BP monitoring program. They viewed the intervention as being timely and believed that it would give them a greater sense of ownership in their care.

*“When you go home with this, the machine for high blood pressure, it’s very good because sometimes you feel like shivering or high blood pressure, you want to check it yourself, you feel like ah! Where will I go to? Maybe it’s night, it’s late. You cannot just go to the hospital at that moment. So, when you have it in the house, you can easily use it any time, any hour. So, no…me I’m saying like it’s one of the best things you people are bringing so far.” (Patient)*

2. *The hospital has a high burden of hypertension disorders of pregnancy and participants perceive the intervention as appropriate, urgent and significant (inner setting and innovation)*

Physicians and nurses expressed an urgent need to address HDP as it is a major cause of mortality among the pregnant patients that present to UATH. Physicians also noted UATH serves as a regional referral centre, making it an ideal setting to implement an intervention that can have wide reach in the community.

*“I can tell you categorically that, those days they used to say that postpartum haemorrhage is the commonest cause of maternal morbidity and mortality. But I will tell you here, the commonest cause of maternal morbidity and mortality is hypertensive disorders.” (Consultant Obstetrician)*

Patient participants felt that improving the management of hypertensive disorders of pregnancy (HDP) was crucial, as it can impact the outcomes for both mothers and their babies.

*“Blood pressure is very, very important during pregnancy because once your blood pressure is being monitored you will know whether your blood pressure is high or low, because high blood pressure is a bad thing during pregnancy which can eventually kill the baby and even the mother who is carrying the baby. So once the BP is being monitored, the doctor will know the medication to give the pregnant woman in order to lower or increase the BP so that there won’t be any complications during pregnancy.” (Patient)*

3. *University of Abuja Teaching Hospital is a tertiary care centre with a multi-disciplinary team of healthcare professionals who have the expertise to provide the necessary care for patients (inner setting)*

Participants believed that UATH has the necessary expertise to care for patients with HDP, due to the presence of a multi-disciplinary team comprised of obstetricians, maternal foetal medicine specialists and cardiologists.

*“We have a referral system here. So, when they (pregnant women) come to antenatal clinic and we find out that the blood pressure is still high, it’s still elevated, we now write a referral to the medical team, they would go there and receive the treatment. But for those that we would admit in the ward, and we are not able to control the blood pressure, we immediately invite the medical team to come in.” (Consultant Obstetrician)*

Patient participants viewed the physicians and nurses as experts in their field, and reported that pregnant patients prefer to receive care at UATH over private hospitals because of the expertise available there.

“*…(T)hey have enough doctors here, specialized doctors. Because if something is happening to you, they will rush you to this place. Even if you go to private hospitals, they are going to refer you here. So, instead of going and they refer you, it’s better you just come here straight.” (Patient)*

Furthermore, participants reported a high level of patient-provider trust which encourages participants to seek care at UATH and return for follow-up appointments. Participants believe that existing relationships with primary and secondary healthcare centres, along with community involvement, can be utilized to facilitate the success of the program.

4. *Protocols are commonly used for other conditions (implementation process)*

Some physicians, nurses, and administrators commented on the use of protocols for managing certain medical conditions including HDP. However, the knowledge of the protocols and their use were inconsistent amongst the participants interviewed.

*“I'm not sure if they have it in the labour ward or the obstetrician (mm-hmm), but I, I don't have a particular protocol that I follow.”* (Family Medicine Consultant)

In addition to protocols, there are ongoing educational talks in antenatal clinics on various conditions that can be leveraged on to improve management of HDP.

*“…(E)very day they come for antenatal, there are health talks that will make them aware. Every, anything they are going through pregnancy, there are health talks that will put them through. But unfortunately, some of these our mothers don't even go for health talk. They don't know. Something that can be prevented, because of ignorance they don't know they will have problems. So, I think if every woman will go for antenatal, it'll go a long way because they are informed and when you are informed, you'll be transformed.”* (Midwife)

5. *Health insurance schemes can be leveraged on to improve access to postpartum care (outer setting)*

Available health insurance schemes can be leveraged to improve access to postpartum care. Participants noted that for patients on the National Health Insurance Scheme, they pay a fraction of the cost and that mitigates the financial barrier for attending clinics.

*“(Patient pays) only 10%, however the insurance covers like 90%.”* (House officer, Obstetrics and Gynaecology)

6. *Mobile technology is widely available and may facilitate implementation of home monitoring program and follow-up via telehealth (outer setting)*

Participants noted that nearly all patients have access to a mobile phone. This will enable follow-up via phone calls and text messages, thereby reducing the number of participants who drop out.

*“… I think quite a number of people do have Android phones now… Quite a good number, at least most of our patients.” (Cardiology Registrar)*

Participants noted that other technological advancements including availability of automated BP devices will simplify the process of implementing a home BP monitoring program.

### Barriers

Participants’ identified key barriers that ought to be addressed to effectively implement and scale up a home BP monitoring program and these include:

1. *Limited knowledge of hypertensive disorders of pregnancy among patients and providers (individual characteristics)*

Participants felt that there was inadequate knowledge and low awareness on HDP and its complications.

*“…because most times we tend to lose these patients in follow-up. That’s one. Because most people don’t think hypertension is an issue until they come down with heart disease or heart failure.” (Midwife)*

Furthermore, overall low literacy levels in the community could pose a challenge for some patients to participate in programs that rely on text messaging because they may have difficulty understanding and using this form of communication.

2. *Pervasive socioeconomic challenges (individual characteristics, outer setting)*

Participants were concerned about the long-term sustainability of the program due to a lack of sustainable funding sources.

*“Because from the top right down into the communities, starting from the government, you need to have them, have policies up there that can support this program. You need to have funding. You need to have, I mean you need to prepare, you have manpower. You need lots of things up there put in place.”* (Family Medicine Consultant)

Some patients may be unable to pay to attend antenatal care or postnatal care clinics and this may affect their ability to enrol and commit to the program. Other trepidations include costs of medication, and any treatment patients may need could impede retention in the program.

*“I think, uh, you should keep in mind the indigent patients. There are a lot of patients here that when we discharge them, they cannot go they will just be around because they cannot pay their bills. Some are there now, they are there*.” (Manager, Maternity Ward)

3.  *Limited transportation networks affect follow-up and retention (outer setting)*

Some patients are referred from remote areas of the Federal Capital Territory at the time of delivery due to maternal or foetal complications requiring a higher level of care. Once they deliver, they may be less likely to return long distances for follow-up appointments due to a lack of transportation and resources. This could affect their willingness to participate in a program that requires them to return for follow-up visits.

*“Most of those, uh, patients that are in the village, even when they discharge them, we'll tell them: please come for your next appointment. We don't get to see them. It's when there's serious issues you will see them. Why didn't you come? "I thought I was fine". They don't used to come. Most times it's the educated ones that will even, we see, but those ones in the village is very, very difficult.” (Nurse, Maternity Ward)*

4. *Inadequate manpower, equipment, and facilities (inner setting)*

Participants lamented on working conditions, highlighting that most hospital wards are understaffed and thus unable to take additional responsibilities. They expressed concerns about the challenges of introducing a new program that would require additional workload for the already burdened workforce.

When asked about the barriers of implementing a program, a nurse manager stated:

*“Manpower number one. manpower, number one; I’m short…in fact the clinic is short-staffed. Secondly there is so much equipment that is not just there, you write drug for patient the, patient will have to go and buy from outside. It’s not all the drugs that are available at the pharmacy, you understand. They’re some things you need, in fact, some instruments are not there.” (Nurse Manager, Antenatal Clinic)*

Physicians highlighted other concerns such as staff burnout, poor staff compensation leading to mass migration of healthcare workers to other countries as factors that could impact successful implementation of the program.

Participants emphasized on inadequate equipment in the hospital, ranging from beds to BP monitors, and more advanced imaging equipment such as CT and MRI scanners. This diagnostic gap limits their ability to care for pregnant women effectively and may impact home BP monitoring program implementation.

Although the hospital has instituted an electronic medical record system, there are some limitations include signal outage and gap in communication between the outpatient and inpatient records, which have not yet been digitized.

5.  *Prevailing religious and sociocultural norms in the surrounding (individual)*

Participants stated that religious beliefs and practices amongst patients can affect enrolment and participation in the program.

*“…another problem we have majorly, our people are so religiously inclined…Medical advice is secondary. Some will tell you that they want to speak to their Imam, their religious leaders. You will be talking to a patient, and they will be calling their pastor or calling a religious leader to pray for them or to seek consent whether they should go ahead with the medical advice.” (Consultant, Maternal Foetal Medicine)*

Some participants also reported that existing traditional beliefs in the community discourage participation in program. Other concurrent and competing family obligations can affect ability of the participants to remain engaged to the program.

6. *Cumbersome processes in hospital wards and clinics (implementation process)*

Patient-participants felt processes in the hospital are too cumbersome especially for pregnant or nursing mothers and involve mothers walking to multiple departments to complete investigations, payments, and appointments. This discourages mothers from attending follow-up appointments in clinics.

*“For us in the tertiary health care centres, I think it’s the rigors of going from one place to the other. It’s so much, it’s so much, erm… how do I put it now? They have a lot of puddles to cross. You have to go get your file, you have to go pay consultation, you have to get a booking, you know…the movement round the hospital could be a little cumbersome. So, sometimes that…that affects them.” (Consultant, Family Medicine)*

**Fig. 1:**
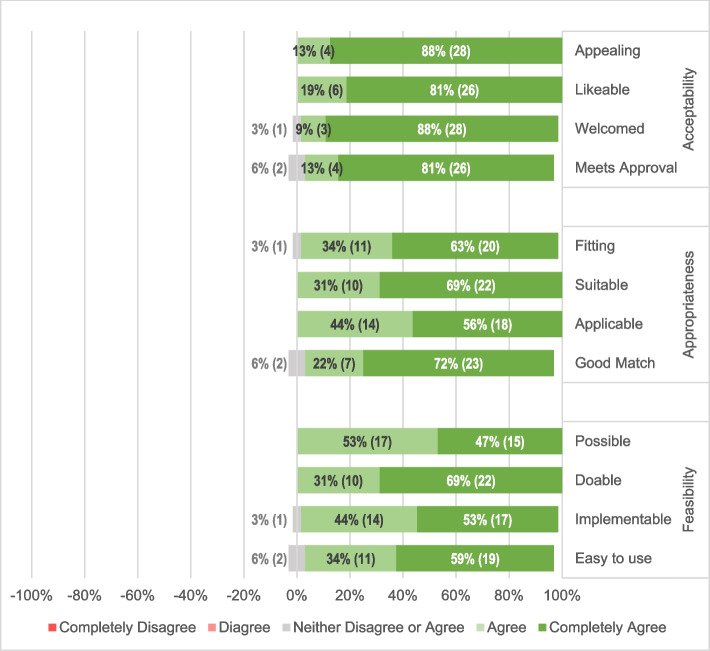
Participants’ Responses to the Acceptability of Intervention Measure (AIM) Survey Assessing the Acceptability, Appropriateness and Feasibility of a Home Blood Pressure Monitoring Program

On the feasibility, appropriateness and acceptability survey, when participants were asked to rate the home BP monitoring program’s feasibility, appropriateness and acceptability using a five-point agreement Likert scale, ranging from *completely agree* to *completely disagree*. 81%-88% *(completely agree)* indicated that a home BP monitoring program would be acceptable, 56%-72% *(completely agree)* reported that it would be appropriate, and 47%-69% *(completely agree)* reported that it would be feasible at UATH. None of the participants disagreed with the notion that the program was acceptable, appropriate or feasible (as shown in Fig. 1).

## Discussion

This qualitative study was conducted as part of a feasibility study to evaluate the factors that can be strengthened to support and the obstacles that need to be overcome to ensure effective postpartum management of HDP with a specific focus on implementing a home BP monitoring program. Key themes that emerged from the interviews and focus group discussions with various individuals who have power or influence over implementation outcomes were summarized.

**Fig. 2:**
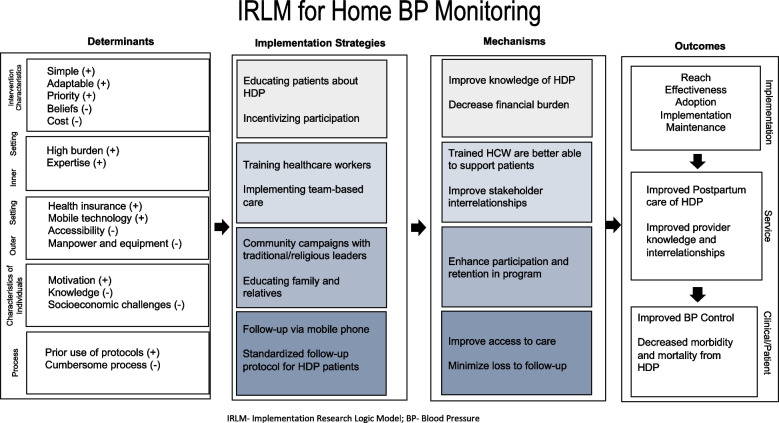
Implementation Research Logic Model for Home BP Monitoring Program

In adapting the innovation for a successful implementation strategy, an implementation research logic model was employed to design the strategies and mechanisms for enhancing the adoption, implementation, and sustainability of a home BP monitoring program as shown in Fig. 2. The adapted program will include strategies to address barriers at the patient, provider, community, and system levels. The strategies discussed are based on the Expert Recommendations for Implementation Change (ERIC) strategies, relevant evidence for hypertension care in pregnant women, and insights from interviewed participants [15].

### Patient-level strategies

Patient education was identified as a crucial strategy at the individual level to enhance understanding of hypertension during pregnancy (HDP), its symptoms, normal and abnormal BP parameters, and potential complications. This approach was recognized by the National Partnership for Maternal Safety's Consensus Bundle and the World Health Organization as a critical aspect for improving maternal outcomes postpartum [10, 16]. In the current study, participants reported that education was important and suggested various delivery methods, including incorporating necessary information into existing prenatal care clinic sessions. They also emphasized that educational programs should start early during prenatal visits to ensure patients are well informed prior to delivery.

A 2019 scoping review found low levels of knowledge among women and healthcare providers regarding cardiovascular complications following HDP in seven countries, including Nigeria [17]. This research highlights the need for widespread investments in education and training as a fundamental strategy to reduce the burden of HDP. Furthermore, a 2022 systematic review on educational interventions aimed at improving knowledge of HDP among pregnant women revealed significant improvements in knowledge, BP control, and overall patient satisfaction, as well as increased awareness of HDP complications [18], demonstrating the feasibility of this strategy.

Another strategy identified at the patient level to enhance health behaviour and address financial barriers in program enrolment and retention was the use of financial incentives. Although healthcare providers favoured cash payments, patients interviewed in this study preferred to receive essential items like diapers as incentives. Use of financial incentives has been widely supported in maternal health research as a means of improving health behaviour [19, 20]. However, there are limited data on the use of financial incentives to improve hypertension care in pregnant women. A randomized controlled trial conducted on patients with uncontrolled hypertension found that financial incentives were effective in achieving short-term BP control, but no sustained effect was observed beyond 3 months of the intervention [21].

### Provider-level strategies

At the provider level, educating healthcare workers on the proper diagnosis and management of HDP at both primary and secondary care facilities can improve early referral and treatment [22]. In tertiary care centres where large multidisciplinary teams are present, communication gaps between providers can be an issue. Formal multidisciplinary rounds or meetings to discuss treatment plans can address this barrier by enhancing stakeholder interrelationships [15]. Team-based care has been suggested as an effective method for delivering postpartum care to women with HDP who have an increased risk of future cardiovascular disease [8].

### Community-level strategies

Community-level strategies encompass involving traditional and religious leaders as respected figures to disseminate information about health programs. Although these strategies are less well-researched, they may be effective methods for tailoring interventions to the local context. A study conducted in Malawi examined the role of traditional rulers in implementing national policies for maternal, new-born, and child health. The findings revealed that these leaders significantly facilitated the implementation process through community campaigns and encouraging women to deliver at healthcare facilities instead of home with traditional birth attendants [23]. Another community-oriented strategy identified by participants who were interviewed was involving and educating the patient's immediate community, including family members, friends, and relatives, to enhance their participation in the program. In an educational intervention study in Nepal, women who were randomized to receiving education with their partners were more likely to attend postpartum visits than women who received education alone [24].

### System-level strategies

Cumbersome processes within the hospital were identified as obstacles that could be tackled at the system level by adapting, tailoring, and standardizing HDP management protocols in the hospital. Other system-level strategies include increasing access to care through utilizing readily available mobile technology for follow-up appointments or providing community outreach clinics and home visits to patients in remote areas. According to a systematic review conducted in 2016, the use of mobile health interventions in low-income countries has been identified as a promising approach to enhance the accessibility and quality of obstetric healthcare services [25].

## Strengths and limitations

The study focused on addressing the critical issue of managing hypertensive disorders of pregnancy (HDP) in Nigeria through the evaluation of a home BP monitoring program employing a comprehensive research approach by utilizing both qualitative and quantitative methods. Through semi-structured interviews, focus group discussions, and the use of the Consolidated Framework for Implementation Research, the study provides in-depth insights into the facilitators and barriers to implementation from various perspectives.

The inclusion of a diverse range of participants, such as healthcare workers specializing in relevant fields and patients, strengthens the validity and applicability of the findings. This study contributes to the literature as it identified facilitators and barriers to implementing a home BP monitoring program specific to the Nigerian context. These findings can inform the development of tailored implementation strategies that address the identified barriers and capitalize on the facilitators, thus increasing the likelihood of successful program implementation. Furthermore, the study's quantitative assessment of acceptability, appropriateness, and feasibility provides valuable insights into stakeholders' perceptions and attitudes, enabling informed decision-making and resource allocation for future implementation efforts.

Although this was the first qualitative study to assess the facilitators and barriers to the implementation of home BP monitoring program in the management of HDP in Nigeria, some limitations must be acknowledged. Despite using a maximal variability sampling method and additional sampling through a snowball approach to reach thematic saturation, there was a possibility of selection bias. The study also did not include interviews with policy makers, community leaders, or family members who could also play a role in the successful implementation of the program. This study’s findings may not be applicable to secondary care centres or other settings as it was conducted at a single tertiary care centre in the Federal Capital Territory.

## Conclusion

The current study demonstrated that a home BP monitoring program was considered acceptable, feasible, and appropriate among physicians, nurses, midwives, administrators and patient groups at UATH. We identified contextually-relevant implementation strategies to leverage facilitators and mitigate barriers that will increase the adoption, implementation, and long-term success of a home BP monitoring program for improved maternal cardiovascular outcomes.

## Data Availability

The datasets used and analysed during the current study available from the corresponding author on reasonable request.

## Abbreviations

AIM: Acceptability of Intervention Measure
BP: Blood Pressure
CFIR: Consolidated Framework for Implementation Research
COREQ: Consolidated Criteria for Reporting Qualitative Research
ERIC: Expert Recommendations for Implementation Change
HDP: Hypertensive Disorders of Pregnancy
SDG: Sustainable Development Goal
UATH: University of Abuja Teaching Hospital
